# Draft Genome Sequence of a Preterm Infant-Derived Isolate of Candida parapsilosis

**DOI:** 10.1128/mra.01273-22

**Published:** 2023-02-27

**Authors:** Steve A. James, Andrea Telatin, David Baker, Rhiannon Evans, Paul Clarke, Lindsay J. Hall, Simon R. Carding

**Affiliations:** a Gut Microbes and Health Research Programme, Quadram Institute Bioscience, Norwich, United Kingdom; b Neonatal Intensive Care Unit, Norfolk and Norwich University Hospitals NHS Foundation Trust, Norwich, United Kingdom; c Norwich Medical School, University of East Anglia, Norwich, United Kingdom; d Ziel–Institute for Food and Health, Technical University of Munich, Freising, Germany; Vanderbilt University

## Abstract

Candida parapsilosis is a human fungal pathogen of increasing incidence and causes invasive candidiasis, notably in preterm or low-birthweight neonates. Here, we present the genome sequence of C. parapsilosis NCYC 4289, a fecal isolate from a preterm male infant.

## ANNOUNCEMENT

Candida parapsilosis is a dimorphic ascomycete yeast belonging to the *Lodderomyces* clade, a large monophyletic group of species, which includes a number of important human pathogens (e.g., Candida albicans) (1). Although often found in the human gut (2, 3), C. parapsilosis is primarily a skin commensal and is present in the hospital environment (4), especially the neonatal intensive care unit (NICU). With a capacity to form persistent biofilms, it can spread by horizontal transmission and is regarded as a significant neonatal pathogen, with low-birthweight preterm neonates at particular risk of infection (5). Here, we combined short- and long-read sequencing to obtain the genome sequence of C. parapsilosis NCYC 4289, a feces-derived isolate from a 31+5-week-old premature male infant delivered by caesarean section. A fecal homogenate was prepared in sterile phosphate-buffered saline (PBS) and cultured on Sabouraud dextrose (SD) agar plates containing penicillin (25 U/mL) and streptomycin (25 U/mL) at 30°C. Species identity, from single colonies, was determined by PCR amplification and Sanger sequencing of the ribosomal DNA internal transcribed spacer (ITS) region of the ribosomal DNA locus using primers ITS1F (6) and ITS4 (7). The ITS sequence of strain NCYC 4289 (GenBank accession number ERZ15609610) is 100% identical to that of the C. parapsilosis type strain CBS 604 (GenBank accession number AY391843).

For short- and long-read sequencing, total genomic DNA was extracted from a stationary-phase SD culture (MasterPure yeast DNA purification kit; Cambio), and cells were treated with Zymolyase (0.25 mg/mL) to aid cell wall disruption with an additional proteinase K treatment step included prior to DNA precipitation. Short-read Illumina sequencing was performed using a modified 20-fold dilution of DNA Prep (Flex) reagent and run on a NextSeq 500 sequencer, producing 7,418,311 paired-end 150-bp reads. Nanopore sequencing was obtained from two methods. First, a novel modified Illumina DNA Prep (Flex) approach used symmetrical 24-base barcoded primers and a long-range polymerase (8). Libraries were pooled and size selected on a SageELF 0.75% cassette, fractions from 4 kb and above were pooled, and long-read sequencing was performed using a MinION sequencer (Oxford Nanopore Technologies [ONT]), ligation sequencing kit SQK-LSK109 (ONT), and flow cell FLO-MIN106 R9.4.1 (ONT). This produced a total of 812,374 reads with an average read length of 4,591 bases. The second method followed the standard ligation protocol using the manufacturer’s recommendations and loading on a second flow cell, producing 852,227 reads (average read length, 4,756 bases). Base calling was performed using Guppy (ONT; v.3.6.0) in high-accuracy mode (model dna_r9.4.1_450bps_hac).

Raw short- and long-read polishing, including the removal of adapters and low-quality bases, was performed using SeqFu 1.16 (9) and fastp 0.23 (10). In addition, long reads of <1 kb in length were discarded. The genome was assembled using Flye 2.9.1 (11), polished with four rounds of Pilon 1.24 (12), and refined using RagTag (13). The genome assembly comprised eight chromosome-sized contigs of >890 kb and a linear mitochondrial genome (29,583 bp). The total size of the genome was 13,082,726 bp, the *N*_50_ value was 2,085,264 bp, and the G+C content was 38.70%. The largest contig in the assembly was 3,026,395 bp. Genome completeness was estimated at 93.0% using BUSCO v5.4.4 (14). Dependencies and scripts are available at https://github.com/quadram-institute-bioscience/ont-candida.

### Data availability.

This whole-genome shotgun project has been deposited at DDJB/ENA/GenBank (BioProject number PRJEB56866 and assembly accession number CAMXCU010000000.1). The version described in this paper is version 1. The raw reads were deposited at SRA (accession numbers ERX9916594 and ERX9917623).
